# A demographic approach for predicting population responses to multifactorial stressors

**DOI:** 10.1093/aobpla/plad023

**Published:** 2023-06-02

**Authors:** Meredith A Zettlemoyer

**Affiliations:** Department of Plant Biology, University of Georgia, 120 Carlton St., 2502 Miller Plant Sciences, Athens, GA 30602-5004, USA

**Keywords:** Antagonistic interactions, demographic modelling, multifactorial, multiple stressor, perturbation analysis, population growth rate, synergistic interactions

## Abstract

Populations face a suite of anthropogenic stressors acting simultaneously, which can combine additively or interact to have complex effects on population persistence. Yet we still know relatively little about the mechanisms underlying population-level responses to multifactorial combinations of stressors because multiple stressor impacts across organisms’ life cycles have not been systematically considered in population models. Specifically, different anthropogenic stressors can have variable effects across an organism’s life cycle, resulting in non-intuitive results for long-term population persistence. For example, synergistic or antagonistic interactions might exacerbate or alleviate the effects of stressors on population dynamics, and different life-history stages or vital rates might contribute unequally to long-term population growth rates. Demographic modelling provides a framework to incorporate individual vital rate responses to multiple stressors into estimates of population growth, which will allow us to make more informed predictions about population-level responses to novel combinations of anthropogenic change. Without integrating stressors’ interactive effects across the entire life cycle on population persistence, we may over- or underestimate threats to biodiversity and risk missing conservation management actions that could reduce species’ vulnerability to stress.

## Teaser

As humans alter multiple facets of the environment simultaneously, addressing multiple, potentially interacting stressors will be critical for predicting species' responses. Yet the effects of multiple stressors across organisms' life cycle has not been systematically considered in demographic studies. Here, Zettlemoyer proposes a framework to explore the role of multiple stressors on population dynamics in order to make realistic predictions about population viability under different conservation scenarios. Specifically, demographic modelling provides a method to integrate stressors' potential additive and non-additive effects across the entire life cycle, provide mechanistic explanations of species loss and spread under combinations of novel conditions, and improve conservation and management plans.

## Introduction

**Figure 1.**
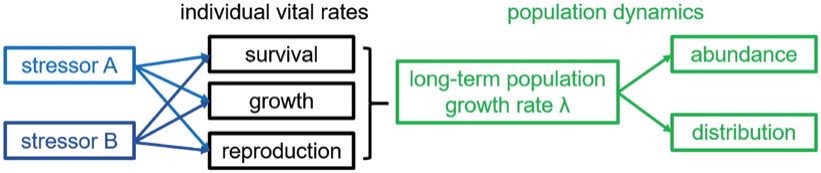
Conceptual approach for predicting population dynamics in response to multiple stressors. Effects on population growth rate (*λ*) depend on the responses of individual vital rates, which can have complex responses to different stressors, and the sensitivity of *λ* to each of those vital rates.

Anthropogenic environmental changes, or **stressors** (Crain *et al.* 2008; see Glossary for bolded terms), such as habitat loss, climate change and invasive species, represent some of the most severe threats to biodiversity worldwide (Urban 2015). These and other stressors can have complex interactive effects (i.e. additive or non-additive) on population persistence (Piggott *et al.* 2015). As humans alter multiple facets of the environment simultaneously, addressing multiple, interacting stressors will be critical for predicting species’ responses (e.g. abundance and distributions) to future environmental conditions (Fig. 1). Studies increasingly document the effects of multiple stressors on individuals, species, communities and ecosystems, yet research on interactive effects of multiple stressors remains relatively rare in population ecology (Capdevila *et al.* 2022; but see Nomoto and Alexander 2021; Zettlemoyer 2022). However, threats rarely impact a population individually (Hernández-Yáñez *et al*. 2016; Wake 2019; Orr *et al*. 2020; Rillig *et al*. 2021) and threat effects may be inconsistent across an organism’s life cycle (Paniw *et al*. 2019; Speißer *et al*. 2022). Therefore, single-threat, single-life-stage approaches can ignore or oversimplify how combinations of stressors ultimately impact species and communities (Capdevila *et al*. 2022; Litchman and Thomas 2022). Ultimately, conserving species will depend on our ability to determine both the underlying population-level processes modulating species’ responses to multiple stressors (Capdevila *et al*. 2020) and which threats have the largest impact on population health (Bernardo *et al*. 2019). Determining threats to long-term population health will require parameterizing **population models** (e.g. matrix population models, integral projection models [IPM]) that quantify the cumulative impact of multiple anthropogenic stressors on population growth rates (Earl 2019).

Most work on population-level responses to anthropogenic stressors has focused on one or more targeted **vital rates** (e.g. survival, growth or reproduction) in size-, age- or stage-structured populations. Reviews report that only 136 studies integrate multiple vital rate responses into plant demographic models (Ehrlén *et al*. 2016) and only 106 studies link climate conditions to multiple vital rates in terrestrial mammals (Paniw *et al*. 2021). However, interacting stressors can have complex effects on different vital rates (Speißer *et al*. 2022), individual vital rates can respond uniquely to stressors (Villellas *et al*. 2015), and vital rates can contribute unequally to overall population growth rates (Ehrlén *et al*. 2016). Therefore, studies that do not integrate across multiple vital rates could under- or over-inflate the importance of an individual vital rate, making predicting the cumulative effects of global change on long-term fitness or population growth difficult. Studies that do not integrate across multiple vital rates could also bias conservation plans, which need to target activities at specific life stages that will benefit population viability (Côté *et al*. 2016; Orr *et al*. 2020). Developing management plans for at-risk species, therefore, needs to consider the non-additive effects of stressors across the entire life cycle. Unfortunately, the long-term demographic data needed for such stage- or age-based population analyses rarely exist (Morris and Doak 2002; Earl *et al*. 2018; Bernardo *et al*. 2018; Johnston *et al*. 2019) despite the fact that population modelling is an essential tool for understanding the effects of multiple stressors on several vital rates and translating those effects into population growth rates (Ehrlén *et al*. 2016).

Here, I argue that demographic studies that consider multiple stressors can partition additive vs. non-additive effects of interacting stressors across the life cycle and allow for more mechanistic explanations of population decline and species loss. Focusing on plant species, I will discuss how (i) interactions among anthropogenic stressors and (ii) counteracting effects of stressors on different vital rates might influence population dynamics. Finally, I outline potential experimental designs that integrate multiple stressor effects across the life cycle to predict extinction risk under future environmental conditions.

## Additive and Non-additive Interactions Among Multiple Stressors

**Figure 2.**
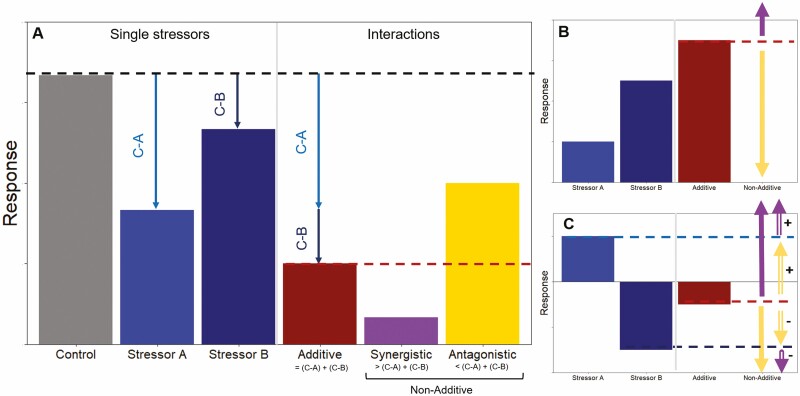
Interactions among stressors can be additive, synergistic or antagonistic. (A) The black dashed line indicates a measurement under a control treatment (grey). The light and dark blue bars and arrows represent responses to single stressors A and B, respectively (left of grey line). An additive response (red bar) occurs when the difference between the control and each stressor (C-A and C-B) is equal to their algebraic sum (indicated with the red dashed line). A synergistic interaction (purple) occurs when the combined effect of two stressors is larger than the sum of their individual effects, while an antagonistic interaction (gold) occurs when their combined effects are less than their sum. Synergistic and antagonistic interactions represent non-additive responses. The additive sum provides a null hypothesis that provides a threshold (red dashed line) for distinguishing the two types of non-additive interactions. (B) When the responses to two single stressors A and B are in the same direction, values below their sum represent antagonistic interactions (gold arrows) while values above their sum represent synergistic interactions (purple arrows). (C) However, these categories become complicated when two stressors induce opposing responses, with some studies considering any value above or below the null synergistic versus antagonistic, respectively, while others advocate for positive or negative classifications of these interactions (double-barred arrow in C, with ± indicating positive vs. negative synergy and antagonism) depending on thresholds based on the single stressor responses (blue dashed lines) (cf. Fig. 2 in Piggott *et al*. 2015).

**Figure 3.**
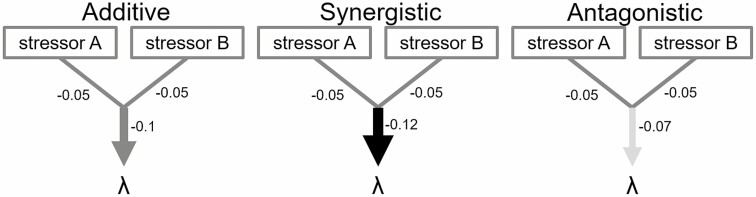
Multiple stressors can have additive, synergistic or antagonistic interaction effects on long-term population growth rates (*λ*). In the case of additive interactions, the combined effects of stressor A and stressor B (medium grey arrow; width of the arrows represents combined effect size) on *λ* equal the sum of their individual effects (medium grey lines). If the interaction between A and B is synergistic, their combined effect on *λ* will be greater than their sum, exacerbating their single effects (black arrow), while if the interaction between A and B is antagonistic, their combined effects will be less than their sum (light grey arrow), negating the potential negative effects of a single stressor.

Understanding how multiple stressors combine to affect biodiversity is an ongoing challenge (Schäfer and Piggott 2018; Speißer *et al*. 2022; Turschwell *et al*. 2022). To date, research has focused on synthesizing studies on single stressors to estimate the possible effects of ecological interactions, but we often detect ‘ecological surprises’ (*sensu*Paine *et al*. 1998) when an interaction has a greater effect than expected (Burgess *et al*. 2021). The expectation is typically **additive effects**, wherein the combined effects of two stressors are equal to the sum of their individual effects (Fig. 2; Crain *et al*. 2008) or when two stressors act entirely independently of each other (Haller-Bull and Bode 2019). For instance, invasion by *Alliaria petiolata* (garlic mustard) interacts additively with deer herbivory to reduce population growth rates of *Trillium erectum* (Bialic-Murphy *et al*. 2019). In these cases, it may be possible to infer the effects of interacting stressors by combining results from single-factor studies. However, stressors can also show **non-additive interactions** wherein the effect of one stressor can change with the intensity of the second stressor (Folt *et al*. 1999; Piggott *et al*. 2015). These non-additive interactions can be **synergistic** when their combined effect has a greater impact than the algebraic sum of their individual impacts (Fig. 2; Darling and Côté 2008; Côté *et al*. 2016). In the invasive thistle *Cirsium vulgare* (common thistle), only the combination of high levels of interspecific competition and insect herbivory results in population decline (Tenhumberg *et al*. 2015). In contrast, non-additive interactions are **antagonistic** when the combined stressor impact is less than their sum (Fig. 2). For instance, the combined negative effects of rising temperatures and more humid climates are less negative than expected based on their individual contributions to survival for two (sub)alpine forbs, *Veronica alpina* (alpine speedwell) and *Viola palustris* (alpine marsh violet) (Tӧpper *et al*. 2018). Altogether, accounting for potential non-additive effects of individual drivers is critical for making quantitative predictions about population-level responses to combined anthropogenic changes (Fig. 3).

Assessing the additive vs. non-additive effects of multiple interacting threats on population dynamics will therefore help prioritize conservation efforts under future combinations of novel conditions. For example, climate warming threatens the viability of a rare forb, *Eurybia furcata* (forked aster), but only when site-level woody encroachment and deer herbivory are high (Bernardo *et al*. 2018). This suggests that management at a local scale (e.g. woody invasive species removal) could reduce this species’ extinction risk under warming. Similarly, invasive species removal is an important management strategy for the rare orchid *Cypripedium candidum* (white lady’s slipper) under moderate climate change scenarios. However, as drought stress increases, protecting groundwater recharge zones in sites near this hydrologically sensitive species becomes increasingly important (Phillips-Mao *et al*. 2016). Both of these studies inform conservation decision-making by assessing multiple interacting stressors and identifying the most important stressor. Applying this process to prioritize management under climate change is critical because while stressors like invasion can exacerbate the negative effects of climate change, their removal provides a ‘low-risk’ short-term management strategy. These low-risk strategies can be important initial actions to reduce the impact of climate change on vulnerable populations (Galatowitch *et al*. 2009), especially given the often-inadequate resources for conservation of rare plant populations (Heywood 2017; Westwood *et al*. 2021).

## Counteracting Effects of Stressors on Different Vital Rates

Understanding responses to stressors across the life cycle can inform which vital rates are most susceptible to a given stressor, but relatively few studies integrate potentially disparate effects of several stressors on multiple aspects of performance into comprehensive assessments of population growth (Ehrlén *et al*. 2016). However, long-term demographic data can be used to identify the specific vital rates altered by a given stressor(s) and whether/how those altered vital rates ultimately influence population growth. Specifically, population models such as matrix projection models or IPMs incorporate the effects of stressors on individual vital rates, which are then used to estimate cumulative population growth rates (*λ*) (Easterling *et al*. 2000; Caswell 2001; Ellner and Rees 2006; Dalgleish *et al*. 2011; Merow *et al*. 2014; Doak *et al*. 2021). Thus, population models can be used to predict *λ* as a function of multiple interacting (a)biotic environmental conditions (Nicolé *et al*. 2011; Adler *et al*. 2012; Merow *et al*. 2017). There are two important considerations here. First, it is important to note that the values of an individual vital rate (e.g. survival ranges from 0 to 1) will not linearly correspond with *λ* values (which can range from 0 to infinity). In order to examine differences in *λ* between multiple stressors and determine the most relevant vital rate, log-transforming *λ* will help interpret the impact of vital rates on *λ* (Orzack and Tuljapurkar 1989). Second, if a study examines multiple stressors, a series of models can be fit against the stressors’ various combinations to then select the most parsimonious model using information criterion approaches (e.g. AICc; Burnham and Anderson 2004; Dalgleish *et al*. 2011) or model averaging (Dahlgren 2010).

Furthermore, **perturbation analyses** (e.g. integrated elasticities and Life Table Response Experiments [LTRE]) can quantify the sensitivity of *λ* to changes in each vital rate and/or environmental variable (van Tienderen 2000). Elasticity analyses decompose direct vs. indirect effects of single vital rates on *λ*; LTREs additionally identify which vital rate(s) drive differences in *λ*. For example, invasion and deer herbivory affect multiple vital rates in *T. erectum* (red trillium), including growth and seedling recruitment, but an LTRE revealed that decreases in fertility largely drive declines in plant fitness (Bialic-Murphy *et al*. 2019). Perturbation analyses can reveal not only which vital rate matters most but also whether a particular stressor influences *λ* at all. In a suite of tallgrass prairie species, an LTRE demonstrated that nitrogen fertilization drives population declines via decreasing survival, but deer herbivory has relatively small effects on individual vital rates and ultimately made no contribution to differences in *λ* (Box 1; Zettlemoyer 2022). Perturbation analyses are critical for predicting responses to multiple stressors because they can capture the demographic mechanisms that drive biological change (Dawson *et al*. 2011; Johnston *et al*. 2019; Schuwirth *et al*. 2019; Otto *et al*. 2020).


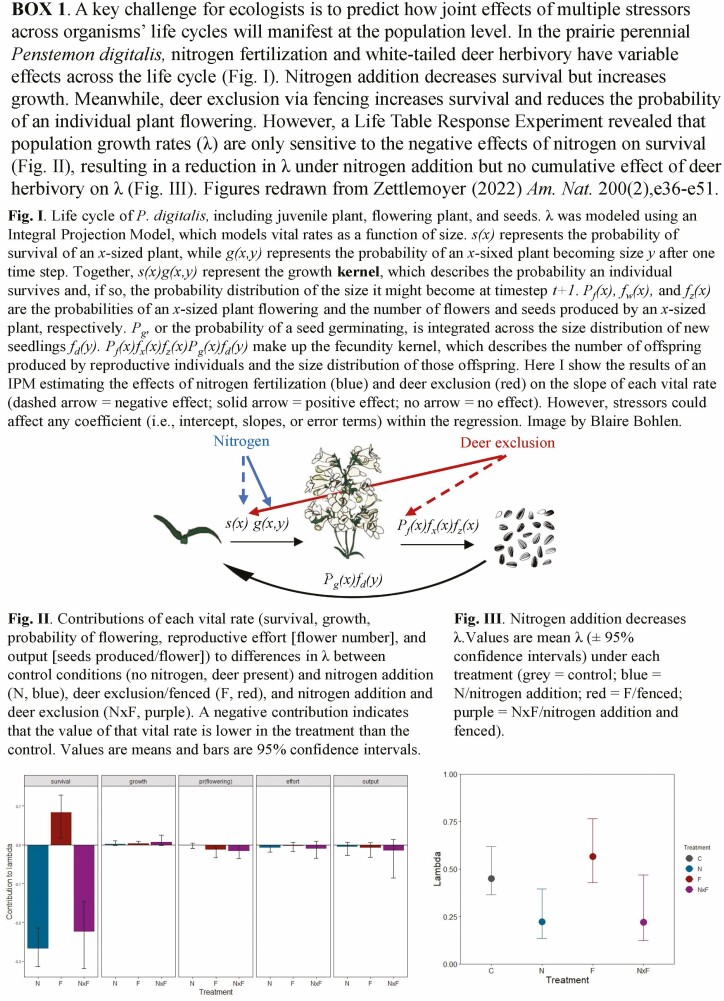


In the two studies above, variation in *λ* was driven by changes in a single vital rate. However, variation in *λ* can also be driven by trade-offs among vital rates (Stearns 1989; Reed *et al*. 2013; Compagnoni *et al*. 2016; Fung *et al*. 2022). Specifically, the combined effects of two vital rates that demonstrate opposing responses to a stressor might result in no net observed effect on *λ* (i.e. **demographic compensation**; Villellas *et al*. 2015). For example, negative covariance between stasis and retrogression alleviates yearly variation in *λ* in *Primula farinosa* subsp. *modesta* (bird’s-eye primrose; Jeong *et al*. 2022). In the alpine plant *Silene acaulis* (moss campion), growth and survival show compensatory responses to warming that buffers overall population growth (DeMarche *et al*. 2018). In this case, the negative effects of lower survival under warming are counteracted by the positive response of growth to warming, resulting in no net effect on *λ*. This approach can be expanded to consider contrasting vital rate responses to simultaneous stressors. In *Phoenix loureiroi* (voyavoy palm), compensatory responses of ramet growth and clonal reproduction buffer populations from the effects of grazing and harvest (Mandle *et al*. 2015). In addition to interactions among individual stressors, **density-dependent processes** can mitigate or enhance stressor impacts at the population level (Moe *et al*. 2005; Dahlgren *et al*. 2014). For example, mortality due to a stressor can reduce competition, such that density-related mortality can compensate for stressor-related mortality (Moe *et al*. 2013). In the weed *Echinochloa crus-galli* (barnyard grass), density-dependent mortality due to post-dispersal seed consumption decreases seedling density, resulting in increased fecundity and therefore compensating for low seedling numbers (Pannwitt *et al*. 2021). In contrast, in *Pityopsis aspera* var. *aspera* (pineland silkgrass), increasing density decreases *λ*, but this effect is alleviated by fire (Gornish 2013). Altogether, understanding how individual vital rate responses scale up (or do not) to consequences at the population level will be critical for conservation in landscapes experiencing multiple simultaneous stressors that can negate or exacerbate their effects at different stages of the life cycle.

## A Need for Added Realism in Modelling Population-Level Responses to Multiple Anthropogenic Stressors

Single-stressor population ecology studies may yield less realistic, if simpler, answers to complex environmental problems (Töpper *et al*. 2018; Litchman and Thomas 2022). Despite their potential to disentangle interactions across life cycles, few demographic studies explicitly link multiple environmental drivers to multiple vital rates across the life cycle (Paniw *et al*. 2021), a concerning limitation given that demographic studies are often aimed at informing management (Crone *et al*. 2012; Earl 2019). I now offer four research priorities aimed at adding realism to studies on multifactorial anthropogenic stressors, including factorial experiments, stressor removal, stressor gradients, and long-term demographic data.

First, multifactorial studies provide the opportunity to investigate the effects of two or more variables simultaneously, a critical future avenue for understanding species’ responses to more realistic present and future conditions (Pazzaglia *et al*. 2022). This approach includes observational studies that take advantage of natural variation in environmental conditions to assess the influence of multiple stressors and experiments wherein stressors are applied while controlling for other influences. The latter represents our best tool for demonstrating causal relationships between stressor and response (Rillig *et al*. 2021), but multifactorial experiments in the field remain rare (Wake 2019; Reich *et al*. 2020). Factorial experiments can be difficult to implement due to the inevitable trade-off between number of replicates and number of treatments. Limited replication can result in low statistical power to detect interactions, defeating the purpose of a factorial design. Meanwhile, investigating a large number of factors results in the ‘combinatorial explosion problem’ (Katzir *et al*. 2019) wherein manipulating a larger number of factors, while more biologically realistic, becomes increasingly difficult due to the rapid increase in the possible number of combinations (although dimension reduction techniques such as Principal Components Analysis can facilitate analysis; Donham *et al*. 2023). Factor number can also influence results; for instance, manipulating an increasing number of global change factors resulted in directional changes in soil properties, but the magnitude of response was variable (Rillig *et al*. 2019). Ultimately, the possibility of non-additive effects (see above) suggests that we cannot rely on a thorough understanding of the effects of single factors to inform us about possible interactions (Norby and Luo 2004). Indeed, increasing the number of anthropogenic factors acting on a community affects plant composition and productivity in ways that differ from expected single-factor effects, suggesting that accounting for multifactorial interactions is crucial for developing a holistic understanding of the consequences of environmental change (Speißer *et al*. 2022).

Multifactorial experiments should also consider two critical differences between current research on multiple stressors and management. First, experimental research often adds stressors to a system, whereas management focuses on removing stressors (Côté *et al*. 2016). Therefore, designing experiments that quantify demographic responses to reductions in key stressors may be more informative for conservation practices. For instance, one approach might be to analyse the effects of invasive species removal efforts in addition to the per-capita effects of invasive species on native abundance or performance (Sofaer *et al*. 2018; Green and Grosholz 2021). The relationship between invasive species abundance and effect (cf. Fig. 1 in Sofaer *et al*. 2018) could affect the optimal allocation of management resources (i.e. the amount of money, time and effort going into eradication programs), especially if invasive species’ impacts on native populations are low until high invasive species densities are achieved. In this scenario, quantifying the effect of low invasive species abundances via removal experiments might avoid over-investment in invasive species control (Yokomizo *et al*. 2009). Second, experimental research often considers global-scale stressors that cannot be alleviated at local scales, so including interactions with manageable local-scale stressors will be useful for on-the-ground conservation efforts. For example, Maxwell *et al*. (2022) considered mechanical thinning to reduce local fire intensity when quantifying the effects of increasing wildfires under future climate scenarios on forest mortality in the western United States, and removal of woody invasives could reduce extinction risk under rising temperatures in *E. furcata* (Bernardo *et al*. 2018). These studies demonstrate that when such local and regional threats interact, local management can reduce species’ vulnerability to broader-scale stressors.

**Figure 4.**
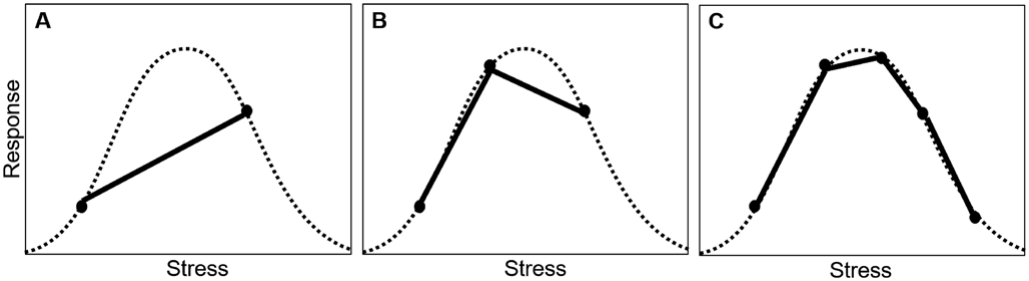
(A) Using only two levels of a stressor (points) may oversimplify the shape of a reaction norm in response to stress (dashed curve), potentially missing biologically meaningful responses to stress, whereas (B) adding even one additional level of a stressor may capture more of the underlying shape. (C) However, five points are considered a minimum to fit nonlinear response curves using a regression design (Collins *et al*. 2022).

Third, identifying the processes underpinning stressor interactions will benefit from experiments with treatments across stress gradients rather than experiments that manipulate the presence/absence of stressors (Jackson *et al*. 2021; Pirotta *et al*. 2022). This can be done in two ways. First, we can examine how performance varies across natural environmental gradients in climate, resource availability or species interactions. For example, Hegland *et al*. (2010) utilized a natural gradient of red deer grazing intensity and a resource gradient to examine the effects of herbivory and resource availability on population dynamics of the boreal shrub *Vaccinium myrtillus*, finding that negative effects of grazing on *λ* were strongest in high-resource environments. While this approach maximizes spatial variation in potential drivers and vital rates, other factors along an environmental gradient may vary in addition to the driver of interest (Ehrlén *et al*. 2016). Alternatively, we can add multiple levels of a stressor to experimental manipulations to better visualize response curves. Reaction norms for most vital rates, if sampled across a range of temperatures, are curved such that vital rates peak at an optimal temperature value, a pattern that is obscured by fitting linear reaction norms between two points (Fig. 4; Arnold *et al*. 2019). These multi-level designs, as well as studies across stress gradients, also allow for regression analyses, which are more robust for estimating multifactorial effects than analysis of variance designs (Collins *et al*. 2022). For example, Merow *et al*. (2017) use regression-based IPM to compare the effects of climate (temperature and precipitation) and microsite conditions (light, nitrogen and pH levels) on the population dynamics of phylogenetically paired native versus invasive species, using a modest number of populations and individuals (*n* = 21 sites and 147 individuals/species). Thus, IPMs, by incorporating environmental covariates in sub-regressions and allowing the use of size-based regression than discrete size classes, can overcome potential pitfalls of underpowered designs or smaller datasets (Ross and Weegman 2022). Moreover, the use of stress gradients can allow tests of correlations between population metrics and a continuous environmental parameter (e.g. a gradient of nitrogen addition rather than nitrogen addition vs. control [Zettlemoyer 2022] or climatic gradients and rankings of habitat suitability [Merow *et al*. 2017]), allowing us to link demography with environmental correlates.

Finally, short-term studies often report a lack of interactions (Komatsu *et al*. 2019), which more often emerge from longer term, more realistic studies (Mueller *et al*. 2016; Komatsu *et al*. 2019; Reich *et al*. 2020; Cusser *et al*. 2021). Additionally, although short-term responses to multiple stressors rarely qualitatively reflect long-term responses (Leuzinger *et al*. 2011), most multistressor studies are conducted over short timeframes (Turschwell *et al*. 2022). More specifically, population ecology studies often focus on a single targeted vital rate (e.g. reproduction) or correlative measures of population size (e.g. species abundance) rather than measuring multiple vital rates across many years (Iler *et al*. 2021), which limits our ability to account for trade-offs in responses to stressors that could limit the effects of individual vital rates on population growth. Additionally, studies that focus on a single vital rate might miss important vital rate responses that will ultimately affect population growth. In the rare endemic plant *Pulsatilla vulgaris* subsp. *gotlandica* (common pasqueflower), temperature and precipitation affect fecundity but not growth or survival (Lindell *et al*. 2022), in contrast to most previous studies linking demography to climate (Morris *et al*. 2020). Measuring only survival or growth would therefore mislead conclusions about weather effects on population dynamics. Understanding how multiple environmental stressors affect long-term population dynamics is a significant challenge in global change ecology, and collecting long-term field data on vital rates across the life cycle will be essential for understanding the interacting effects of global change on demography (Clutton-Brock and Sheldon 2010; Johnston *et al*. 2019; Paniw *et al*. 2021).

## Conclusion

Interactions among stressors will continue to generate uncertainty and surprises in ecological research (Côté *et al*. 2016; Burgess *et al*. 2021), but synergisms and antagonisms are commonly documented in both experiments and natural systems (Murdoch *et al*. 2020). Determining how multiple stressors interact will be key to attempts to mitigate their effects. However, studies also need to systematically consider how multiple stressor effects on demographic parameters across the entire life cycle will scale up to affect population- and community-level patterns (Moe *et al*. 2013; Haller-Bull and Bode 2019; Simmons *et al*. 2021). Multifactorial demographic studies that integrate responses across organisms’ life cycles will allow us to better predict long-term responses to co-occurring anthropogenic changes, particularly if the goal of such studies is to inform conservation and management efforts under future environmental conditions.

## Glossary

**Stressors:** Aiotic and biotic environmental factors that exceed their natural range of variation due to human activity.

**Population models:** Type of mechanistic model that links individual state variables to changes in population growth, density and structure over discrete time steps; types of population models include (i) matrix population models, which use stage- or age-structured state variables and (ii) IPM, which use continuous (often size-dependent) state variables to predict population growth rates as well as (iii) population viability analyses that estimate population size (e.g. count data) from one monitoring event to the next.

**Vital rates:** Rates corresponding to an organism’s individual life stages or progression through development (e.g. birth/germination, growth, survival, reproduction, mortality).

**Additive effects:** Type of interaction when the combined impact of two or more stressors on an ecological response is equal to than their algebraic sum; in this case, studies of single stressors can be added to estimate their combined effect.

**Non-additive interactions:** Type of interaction when the combined impact of two or more stressors on an ecological response is not equal to their algebraic sum, such that the intensity of response to one stressor changes with the presence of the other; can be synergistic or antagonistic.

**Synergistic:** Type of non-additive interaction when the combined impact of two or more stressors on an ecological response is greater than their algebraic sum; one stressor can exacerbate the effects of another.

**Antagonistic:** Type of non-additive interaction when the combined impact of two or more stressors on an ecological response is less than their algebraic sum; one stressor can alleviate the effects of another.

**Perturbation analyses:** Demographic analyses exploring how population growth rates respond to changes in vital rates; types of perturbation analysis include (i) prospective analyses (e.g. sensitivity and elasticity) that test how much *λ* depends on vital rates independent of variability and (ii) retrospective analyses (e.g. LTRE) that decompose variance in *λ* as a function of variance in vital rates.

**Demographic compensation:** Opposing vital rate responses to stressors wherein the positive effects of one vital rate can negate the negative effects of another.

**Density-dependent processes:** Population-level processes (e.g. vital rates) that are sensitive to fluctuations in population density (i.e. the number of individuals per unit area).

**Kernel:** A probability density function that maps the change in the size distribution of individuals at time *t* to the size distribution after one timestep (*t +* 1).

## Data Availability

No new data were generated or analysed in support of this research.

## Notes

**Populations and Communities. Chief Editor:**
